# Evaluating the Impact of Kefir Consumption on Dental Caries and Periodontal Disease: A Narrative Review

**DOI:** 10.3390/dj13020086

**Published:** 2025-02-18

**Authors:** Anna González-Rascón, Elda Georgina Chávez-Cortéz, Angélica Hurtado-Camarena, Nicolás Serafín-Higuera, Sandra Castillo-Uribe, Víctor Manuel Martínez-Aguilar, Bertha Arelly Carrillo-Ávila, Viviana Pitones-Rubio

**Affiliations:** 1 Facultad de Odontología, Universidad Autónoma de Baja California, Mexicali 21040, Mexico; gonzaleza1@uabc.edu.mx (A.G.-R.); hurtadoa@uabc.edu.mx (A.H.-C.); nserafin@uabc.edu.mx (N.S.-H.); sandra.castillo@uabc.edu.mx (S.C.-U.); 2 Facultad de Odontología, Universidad Autónoma de Yucatán, Mérida 97000, Mexico; elda.chavez@correo.uady.mx (E.G.C.-C.); victor.martinez@correo.uady.mx (V.M.M.-A.); arelly.carrillo@correo.uady.mx (B.A.C.-Á.)

**Keywords:** kefir, dental caries, probiotics, microbiota, periodontal diseases, biofilm, oral health

## Abstract

**Background/Objectives**: Dental caries and periodontal diseases are the most common illnesses in the oral cavity and represent a public health concern globally. In recent decades, diverse studies showed that Kefir, a traditional beverage that can be milk- or water-based, contains a complex microbial community and has health benefits. The goal of this review was to update the current knowledge of kefir consumption and its impact on oral health. **Methods:** The search of a combination of keywords—kefir; dental caries; probiotics; microbiota; periodontal diseases; biofilm; and oral health—was conducted using PubMed, Google Scholar, and Web of Science databases for studies in human subjects. **Discussion:** The research suggests that kefir consumption may aid in decreasing counts of microorganisms typically associated with oral illness. **Conclusions:** Kefir has the potential to inhibit certain oral pathogens and reduce biofilm formation by promoting diversity within the oral microbiota, suggesting that kefir could be a promising adjuvant treatment for dental caries and periodontal diseases by improving oral health.

## 1. Introduction

Oral health is a critical component of overall well-being, with dental caries and periodontal disease being among the most prevalent conditions affecting the oral cavity. Dental caries, commonly known as cavities or tooth decay, result from the demineralization of tooth enamel by acids produced during the bacterial fermentation of dietary carbohydrates [1]. According to the Global Oral Health Data Bank, the incidence of dental caries ranges from 49% to 83% globally. Similarly, the World Health Organization (WHO) estimates that nearly 100% of the global population experiences dental caries at some point, highlighting its near-universal prevalence [2]. Among children, dental caries is a major public health concern, affecting approximately 50% of preschool-aged children worldwide [3].

Periodontal disease is an inflammatory condition involving the gums and supporting structures of the teeth, often resulting from plaque accumulation. This condition can be exacerbated by factors such as poor oral hygiene, diabetes, obesity, and hormonal changes during pregnancy, among others [4]. Between 1990 and 2019, the prevalence and number of cases of severe periodontal disease increased significantly. The number of cases grew by nearly 540 million, representing a 24% increase in prevalence. Upper-middle-income countries experienced the largest rise in prevalence at 35%, while low-income countries saw a 130% increase in case numbers. In contrast, high-income countries had the smallest rise in case numbers, at 51% [5].

Both conditions are interrelated, as poor oral hygiene and the presence of pathogenic bacteria can exacerbate both caries and periodontal disease. Sugar consumption and rapid digestion of sucrose, fructose, and glucose, make biofilms very stable to bacterial species that can tolerate low pH and adhere to oral surfaces [6,7]. One study reported that antibiotics such as erythromycin, metal salts, and fluoride have been used against cariogenic bacteria [8]. However, these medications have been linked to side effects such as tooth discoloration and discomfort. In periodontal disease, treatments are often a combination of clinical and pharmacological strategies tailored to the individual needs of the patient to effectively manage and control periodontal disease. It is necessary though to do a deep cleaning procedure that removes plaque and tartar from below the gumline to promote healing and reduce bacterial accumulation [9,10].

Effective oral health management relies on well-established practices such as plaque control and healthy dietary habits. Additionally, probiotics are being explored as a potential adjunctive therapy in dentistry. These microorganisms have shown promise in preventing and treating biofilm-related oral diseases, including dental caries, periodontal disease, oral soft-tissue lesions, candidiasis, and halitosis [2,8,11].

Kefir is a fermented beverage containing various probiotic bacteria and yeast strains that differ based on its starter (kefir grains) and fermentation substrate, which is typically dairy. These variations give kefir distinct organoleptic and nutraceutical properties, such as immunostimulation and antimicrobial, antiallergenic, and anticancer activities [12,13,14]. Like other fermented foods, kefir’s potential to promote oral health is thought to arise from its ability to balance the complex oral microbiome, due to its unique microbial composition, including Lactobacillus and Bifidobacterium species. These microorganisms help maintain oral microbiota balance by producing organic acids and antimicrobial compounds. Research indicates that short-term consumption of probiotic-rich foods like kefir can elevate salivary pH, reducing the risk of dental caries [15]. Kefir’s probiotic effects can also significantly lower salivary levels of *Streptococcus mutans*, with results comparable to sodium fluoride in young adults [16]. Moreover, daily kefir consumption, combined with probiotic toothpaste, has been linked to reduced microbial colonization in orthodontic patients [17].

These findings underscore its potential as a therapeutic and preventive supplement for caries prevention and gum health. The incorporation of probiotics into dental care not only modernizes traditional practices but also highlights the significance of the oral microbiome. By investigating the interactions between microorganisms and oral health, researchers can formulate targeted strategies for improved prevention and treatment, ultimately leading to better overall outcomes. This review aims to analyze published reports on the effects of kefir consumption on the prevention and treatment of caries and periodontal disease.

## 2. Methodology

The methodology involved the search of a combination of keywords—kefir, dental caries, periodontal diseases, probiotics, microbiota, biofilm, and oral health—using PubMed, Google Scholar, and Web of Science databases for studies in human subjects. The keywords were searched with MeSH terms.

## 3. Probiotics

WHO and the Food and Agriculture Organization (FAO) describe probiotics as live microorganisms that, administered in adequate amounts, confer health benefits on the host [18]. Probiotics are considered a bacteriotherapy that can control the ecology of the oral cavity by reducing the number of pathogenic bacteria, becoming a strong promising alternative [12,16,18]. Several studies describe the beneficial effects of probiotics in the host, such as inhibiting pathogenic bacteria, modulating gut microbiota, and suppressing low-grade systemic inflammation [19].

Probiotics can exert indirect and direct mechanisms to aid in the treatment of inflammatory diseases of the oral cavity. Indirect mechanisms include effects on local and systemic immunity, regulation of mucosal permeability, and competition of colonization sites. Direct mechanisms refer to the inhibition of plaque formation, competition for nutrients, and production of antipathogenic compounds [20]. These effects are generated due to different mechanisms of action such as increased adhesion to the intestinal mucosa, production of antibacterial substances, competitive exclusion of pathogens, simultaneous suppression of bacterial adhesion, and modulation of the immune system [21].

Probiotics-mediated bioconversion in periodontitis involves beneficial bacteria transforming compounds to enhance oral health. These probiotics can convert dietary and endogenous molecules into antimicrobial and anti-inflammatory metabolites, reducing harmful pathogens and inflammation in the oral cavity. By influencing both microbial balance and the host’s immune response, probiotics support periodontal health, making them a promising adjunctive treatment for managing periodontitis [22].

Probiotics are consumed as a nutritional supplement, prophylactic treatment, or functional food, and they play an important role in the prevention and treatment of oral diseases such as dental caries, periodontitis [23], halitosis [24], candidiasis [25], and gingivitis [26,27]. Table 1 summarizes some species of bacteria and yeasts used for oral probiotic preparations. In this sense, milk and water kefir are beverages with a probiotic composition that could be used as a functional food because of the oral health benefits they confer [13,28,29].

## 4. Milk Kefir or Dairy Kefir

The word kefir comes from the Turkish word kef, which means “sweet taste”. Milk kefir is a fermented beverage originally produced for centuries in the Caucasus Mountains by mixing milk and kefir grains. These grains are gelatinous granules that are around 1–6 mm in diameter but can measure up to 15 mm and range in size from 0.1 to 4 cm in length. They have an irregular, rough, and convoluted surface that looks like a cauliflower floret of a white or cream color [13,19,28]. Kefir grains contain bacteria and yeasts responsible for milk fermentation, such as lactic acid bacteria or LAB (*Lactobacillus* spp., *Lactococcus* spp., *Leuconostoc* spp., *Pediococcus*, and *Streptococcus* spp.) and yeasts (*Candida* spp., *Saccharomyces* spp.) which are mixed with milk and sugar in a polysaccharide matrix named kefiran to make kefir drinks [28,30].

Studies demonstrated that milk kefir inhibits gram-negative bacteria such as *Salmonella typhi*, *Escherichia coli*, *Pseudomona fluorescens*, and *Pseudomonas aeruginosa*. On the other hand, kefir-derived bacteria or structural components isolated from kefir inhibit gram-positive bacteria such as *Listeria monocytogenes*, *Staphylococcus aureus*, *Bacillus subtilis*, and *Enterococcus faecalis*. The proposed mechanism is increased membrane permeability that disrupts bacterial cells leading to cell death [13].

In recent years, research has been reported on milk kefir or its bioactive compounds that induce health-promoting effects through in vitro or in vivo studies, showing several biological activities like antimicrobial, anticancer, antihypertensive, anti-inflammatory, antidiabetic, and antiallergenic activity, as well as antioxidant properties and strengthening of the immune system function [13,28,35,36,37,38,39,40,41]. In humans, more recent research has shown beneficial effects on the intestinal homeostasis of COVID-19 patients after consumption of kefir; it can improve inflammatory factors slightly but does not improve the symptoms of the disease [42]. However, there is little evidence of the oral clinical effects of kefir in human health, and most studies have been conducted in one geographical area in Asia and Europe, as can be observed in Table 2.

## 5. Water Kefir or Non-Dairy Kefir

The origin of water kefir is still unknown; it is believed that initial grains came from an Opuntia fig plant [28,47]. Guzel [28] reported that grains of water kefir are typically smooth and rarely have visible sub-unit granules as observed in milk kefir. The grains are gray in color and were described as “rock salt”, but this color depends on the fruits or vegetables used for the fermentation process [28,48]. Water kefir grains range in size from 5 to 20 mm in diameter [47]. Bacteria responsible for generating a matrix of polysaccharides (mainly dextran and levan in minor proportions) that contains microbiological complexes of water kefir grains include lactic acid bacteria such as *L. casei*, *Leuconostoc mesenteroides*, *L. nagelli*, *L. hordei*, and *L. hilgardii*, acetic acid bacteria, and yeasts [47,49].

Water and milk kefir, though similar in their probiotic benefits, differ significantly in their microbial composition due to the substrates used in fermentation. Water kefir has 70% *Lactobacillus* sp., 10% *Leuconostoc* sp., 10% *Acetobacter* sp., 5% *Bifidobacterium* sp., and 5% other bacteria approximately, and milk kefir has 50% *Lactobacillus* sp., 20% *Leuconostoc* sp, 10% *Streptococcus* sp., 8% *Pediococcus* sp., 7% *Lactococcus* sp., and 5% other bacteria approximately [28]. These bacterial populations contribute to kefir’s antimicrobial activity and probiotic properties such as non-pathogenicity, tolerance to gastrointestinal conditions, adhesion to the gastrointestinal mucosa, ability to colonize, and competitive exclusion of pathogens [49]. However, the specific strains and their interactions vary between water- and milk-based fermentations, impacting the beverage’s health benefits and flavor profiles [28,50].

In vitro or in vivo studies of water kefir have also reported antimicrobial activity against *Candida albicans*, *Salmonella typhi*, *Shigella sonnei*, *Staphylococcus aureus*, and *Escherichia coli* [51]. For immunomodulatory and anti-inflammatory activity, there are a few studies; however, the outcomes show that water kefir helped produce a controlled inflammatory response [52,53]. One study reports more antioxidant properties of water kefir and an increase in dextran through the production of bioactive compounds due to longer fermentation times [54]. Antihyperglycemic effects are reported on strains isolated from water kefir [55].

## 6. Polymicrobial Community

The oral cavity harbors a wide diversity of microorganisms that form the oral microbiota, and homeostasis in host–microbiota interactions lead to a healthy periodontium. These microorganisms are very important mediators of oral health and disease, along with other clinical and risk factors [56]. Dental plaque is a polymicrobial community and can exert inter-reign communication with eukaryotic cells, therefore influencing local and systemic homeostasis and impacting health status and disease development [2,57].

In dental caries, some examples of acid-producing cariogenic communities include *Lactobacillus* spp., *Bifidobacterium* spp., *Actinomyces* spp., *Propionibacterium* spp., *Corynebacterium* spp., *Granulicatella* spp., and *Scardovia* spp. [58,59]. Some bacterial species such as *Streptococcus mutans* and *Streptococcus sobrinus* are key matrix producers in cariogenic biofilms, and their metabolic products can signal and benefit the proliferation of other pathogens that can also enrich the cariogenic microenvironment [8].

Socransky et al. (1998) [60] identified four microbial complexes based on their color associations: the red complex, linked to periodontal disease; the yellow complex, associated with periodontal health; and the orange complex, recognized as periodontal pathogens. The bacteria of the red complex—*Tannerella forsythia*, *Porphyromonas gingivalis*, and *Treponema denticola*— are now recognized as primary pathogens driving advanced periodontal disease. Recent research has expanded our knowledge, revealing a more complex landscape on how periodontitis results from polymicrobial synergy and dysbiosis that disturbs the ecologically balanced biofilm associated with periodontal tissue homeostasis [4].

In 2019, Abu Fanas et. [61] reported the relative abundance of novel periodontal pathogens and bacterial complexes in Stage II generalized periodontitis. The findings confirm that the microbial complexes present in periodontitis reflect an established dysbiotic environment. Early colonizers, such as *Streptococcus* species (yellow complex), adhere to the acquired pellicle and provide a foundation for secondary colonizers like *Actinomyces naeslundii* and *Veillonella atypica* (purple and green complexes). These early microbial communities pave the way for intermediate colonizers, notably *Fusobacterium nucleatum*, which create anaerobic conditions conducive to the red complex bacteria—primary pathogens driving advanced periodontal disease. This progression, from a symbiotic oral microbiota to incipient dysbiosis in gingivitis and ultimately frank dysbiosis in periodontitis, underscores the destructive immune response leading to tissue damage. These findings emphasize the importance of understanding microbial interactions and their impact on periodontal health, offering avenues for targeted interventions and improved therapeutic strategies.

## 7. Dental Caries

Dental caries is associated with changes in the composition of biofilm due to dietary carbohydrates and other host factors that cause the proliferation of pathogenic bacteria and increase lesions in enamel caused by acidogenic and aciduric microorganisms [2,58,62,63]. The evidence points to acidogenic species that can survive by colonizing buccal epithelial cells of the tongue’s dorsum, where they serve as reservoirs for supragingival and subgingival plaque [1,7,63]. The biofilm starts by coating tooth surfaces with salivary glycoproteins, and then species like *S. mitis* and *S. mutans* produce a layer of exopolysaccharides (EPSs) and acidic metabolites that facilitate the adherence of other microorganisms and induce dental caries [2]. The production of EPSs provides binding sites for adhesion to the tooth surface by generating glucans through glycosyltransferases (Gtf B, Gtf C, and Gtf D), and this binding of bacteria to glucans occurs through the proteins GBpA, GBpB, GBpC, and GBpD. Studies have reported that GBpB and GBpC are specific glucan receptors and therefore play an important role in the adhesion of microorganisms and the formation of biofilms [47]. Studies have reported the presence of *Candida albicans* in the plaque of toddlers, where *C. albicans* interacts with *S. mutans* to colonize tooth surfaces due to exoenzymes secreted by *S. mutans,* which bind to Candida surfaces synthesizing glucans that form mixed biofilms, indicating that polymicrobial communities can act in synergy, triggered by host dietary sugars levels, which can be exacerbated by other factors [58].

Oral sucrose-dependent bacteria are prone to fermenting sugar from diets, and to produce acids, lowering the pH of the biofilm fluid to 5.0 or lower. Acidification causes a shift and disruption in the microbial community and the tooth-enamel mineral homeostasis. Consequently, tooth minerals are dissolved, and this promotes the proliferation of cariogenic bacteria in the biofilm [12,62]. For the above, maintenance of pH is important in the oral ecosystem and can be achieved with low and infrequent sugar consumption, and the remotion of biofilm that allows microbial communities to remain stable, because the pH decrease is neutralized by saliva and prolonged acidification states are avoided [58].

Besides nutrient availability, there are other microbial-associated risk factors like the transfer of resistance genes, complexity of the matrix, and physical protection provided by EPS [2]. Additionally, other factors contribute to the development of dental caries, for example, poor oral hygiene, changes in saliva, and inadequate exposition of fluoride [58]. Alterations in the oral microenvironment generated by bacterial fermentation can affect the balance of Ca^2+^, (PO_4_)^3+^, and F ions, contributing to the onset and progression of dental caries. The acid produced by sucrose fermentation binds to Ca^2+^ and (PO_4_)^3+^, causing tooth demineralization and the development of a caries lesion.

## 8. Periodontal Diseases

In periodontal disease, the formation of biofilms on the oral surfaces triggers an inflammatory response to microbial antigens recognized as danger signals. These biofilms consist of microorganisms that promote a dysbiotic oral environment, leading to a dysregulated and destructive immune response [64]. Gingivitis, an inflammatory condition affecting the gingival epithelium and connective tissue, is a significant risk factor and precursor to periodontitis. Chronic periodontitis, on the other hand, is characterized by the destruction of the gingival tissue attachment to the tooth, formation of periodontal pockets, degradation of the periodontal ligament, and loss of alveolar bone [4]. Risk factors for periodontal diseases include systemic conditions such as diabetes, obesity, smoking, unhealthy diets, stress, and poor oral hygiene [65].

## 9. Discussion

In recent decades, research in oral health has included clinical trials with probiotic products as a preventive alternative for caries and periodontal disease. Beverages have shown promising benefits for lowering the salivary bacteria count and can be used to prevent enamel demineralization [12,43]. Probiotics promoting a neutral pH environment prevent enamel demineralization and support the remineralization process, further contributing to oral health maintenance [30].

The proposed mechanism by which probiotics support oral health involves competitive exclusion, where probiotics outcompete harmful bacteria for adhesion sites on tooth surfaces, and the production of bacteriocins and hydrogen peroxide contained in kefir, which directly inhibit pathogenic microorganisms [43,66].

On the other hand, the consumption of probiotic-containing yogurt in infants has benefits for oral health like buffering capacity that reduces the caries condition. Yogurt and kefir have been shown to inhibit GTF activity and reduce the release of fructose, a by-product of GTF action on sucrose. This suggests that both dairy products may have potential as caries-preventive agents due to their ability to disrupt the cariogenic activity of *S. mutans* [67,68].

The balance between microorganisms plays a critical role in maintaining oral homeostasis. Fermented beverages like kefir, which are rich in probiotics, may help sustain eubiosis—a state of balance in the oral microbiome where symbiotic and commensal relationships are preserved under healthy conditions [69].

The use of probiotics as a complementary therapy targeting and regulating destructive inflammation is gaining attention. Their regulatory potential lies in promoting balance within the oral microbiome and reducing inflammation, thereby complementing traditional treatments [9,10]. This approach is particularly valuable for patients predisposed to a chronic hyperinflammatory response due to genetic, systemic, or environmental factors, where conventional therapies alone may be insufficient. Recognizing the limitations of traditional treatments underscores the need for innovative strategies, such as kefir consumption, to address periodontal disease effectively [70].

Long-term kefir consumption can significantly impact systemic health by supporting gut health, reducing inflammation, and improving metabolic and immune function. Kefir enhances the diversity of beneficial gut microorganisms like Lactobacillus and Bifidobacterium, which strengthen the intestinal barrier, improve digestion, and help maintain a balanced gut microbiome [71]. Its immunomodulatory and anti-inflammatory properties, driven by bioactive compounds, aid in managing systemic inflammatory conditions and reducing the risk of metabolic disorders like type 2 diabetes and cardiovascular disease [29]. Additionally, kefir has shown potential in regulating lipid profiles, promoting weight management, and influencing the gut–brain axis, which may benefit cognitive function and mood regulation [71]. These systemic effects make kefir a valuable dietary addition for overall health.

Table 2 summarizes recent studies examining kefir’s effects on caries prevention and periodontal disease in human subjects. These studies consistently highlight the protective and preventive role of milk kefir, while evidence for water kefir remains unreported. Three studies focused specifically on children and adolescents, applying consistent inclusion criteria such as prior knowledge of oral hygiene techniques and clear oral health indicators at the baseline [17,44,45]. Findings indicate a reduction in various cariogenic bacterial species. Of these studies, only two specified the kefir dosage, with protocols differing between them; notably, the study with the most significant results combined kefir use with toothpaste. Given kefir’s polymicrobial nature, however, it is unclear whether its effects derive from specific probiotic strains or synergies within the microbial community [17]. One study relied on questionnaire responses rather than defined intake quantities, potentially introducing bias [44]. Additionally, a comparative study using a sodium fluoride mouth rinse found that kefir’s effectiveness in reducing salivary *S. mutans* species was comparable to the mouth rinse; this study focused on patients aged 22–32 [28]. The results from Alp et al. and Çoğulu et al. [17,43] suggest that kefir’s effectiveness against cariogenic pathogens may be enhanced by a higher microbial diversity, and importantly, was also dose-dependent, with increased kefir amounts yielding better outcomes.

For caries prevention, it has been reported that milk products have a buffering capacity that inhibits the dental caries process [45]. Furthermore, sheep milk used for fermentation is better for the generation of probiotic cultures and bioactive peptides that influence enamel demineralization [72,73]. The viscous consistency of milk kefir provides adherent properties, remaining in the oral cavity for a long time and attaching to the tooth enamel [17,43]. Several biochemical reactions occur during the fermentation process of kefir in the oral cavity. In this process, enzymes like invertases or hydrolases are produced and reduce sugars and lipids, producing different metabolites and increasing microorganism proteins [74].

**Figure 1:**
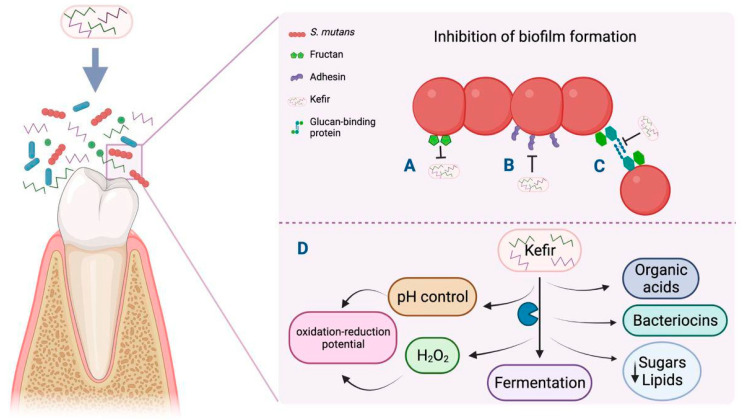
Antibiofilm mechanism triggered by kefir in dental caries through inhibition of carbohydrate metabolism. Created with Biorender https://biorender.com/k37q453 (accessed on 24 November 2024).

Although kefir’s mechanism of action against dental caries is unknown, it is proposed that kefir can balance the oral ecosystem due to its probiotic strains. This allows the proliferation of a bacterial film through diverse mechanisms, such as the production of antimicrobial substances like bacteriocins, hydrogen peroxide, and organic acids that antagonize pathogens such as *S. mutans* for adhesion sites at the mucosa. The metabolic products of microorganisms that show LAB and yeasts from multi-strain kefir regulate plaque pH and produce an oxidation–reduction potential that establishes unfavorable conditions for pathogen development (Figure 1) [43,74].

In vitro studies mention that Lactobacilli strains isolated from milk kefir showed potential against caries-reducing biofilms in the oral cavity due to the effectiveness of biofilm formation-associated genes. Recently, *L. kefiranofaciens* was isolated from kefir; it inhibits *S. mutans* growth through changes in the gene expression of genes associated with biofilm formation and virulence factors related to carbohydrate metabolism. Gene downregulation causes a decrease in fructan production, biofilm formation genes, and adhesion genes encoding binding proteins like glucans and adhesins (Figure 1) [8]. Early clinical studies have reported that the oral administration of *L. paracasei* in a short-term intervention showed inhibitory effects on colony-forming units (CFUs) from *S. mutans* [75] and *L. rhamnosus* and, in milk powder or fermented form, could reduce CFUs from cariogenic *S. mutans* [76]. Altogether, the detailed study on the mechanisms and effects of kefir in dental caries is relevant for new anti-cariogenic therapeutics strategies.

On the other hand, Diabetes Mellitus is a comorbidity of periodontal disease also affected by systemic inflammatory dysregulation, where glycemic control is affected. There are reports of anti-hyperglycemic activity from strains isolated from water kefir [55], which opens the discussion to consider testing water kefir properties in a properly designed clinical assay with non-vulnerable patients.

There are not many clinical assays evaluating the effects of kefir consumption on gum health. Nevertheless, the adjunctive use of probiotics can effectively inhibit oral pathogens and improve key clinical indices related to periodontal health such as the plaque index, gingival index, bleeding on probing, periodontal pocket depth, clinical attachment loss, and gingival crevicular fluid volume. Furthermore, probiotics may also help promote immunoregulation, thereby offering a multifaceted approach to managing periodontal disease and enhancing long-term clinical outcomes [77].

While there is limited metagenomic research on the impact of kefir on oral microbiota, studies have demonstrated its significant influence on gut microbiota. Kefir contains high concentrations of lactic acid bacteria, ranging from 8 to 10 log CFU/mL, and these bacteria exhibit excellent survivability and colonization abilities within the host gut. In fact, research by Hamet et al. (2016) [78] reported that kefiran displayed a bifidogenic effect in an animal model, suggesting that kefir-derived compounds can enhance the indigenous Bifidobacterium population. While the effects of kefir on the oral microbiome remain underexplored, its ability to positively influence the gut microbiota highlights the potential for similar effects on the oral cavity, warranting further investigation into the by-products of kefir and its effects on oral health.

Kefir’s use as an adjunct treatment faces several challenges, primarily production and dosage standardization. Its composition varies depending on the origin of the grains, fermentation substrate, and processing methods, resulting in variations in probiotic strains and concentrations. Additionally, kefir’s live microorganisms are sensitive to storage conditions, which may affect its stability [28]. Individual variability in microbiota composition, as well as cultural and dietary restrictions, can influence its efficacy and put immunocompromised patients at risk. These challenges must be addressed to realize kefir’s full potential as an adjunct treatment.

Determining an appropriate dosage is unclear, with factors like frequency, volume, and duration requiring further study. Bessa et al. published a scoping review about Kefir as a therapeutic agent in clinical research. The reported dosage of kefir in various studies typically ranged from 100 mL to 500 mL per day, consumed over a period of several weeks to months, depending on the health outcome being studied. We saw the same ranges in Table 2. This range allows for flexibility in intake while still delivering sufficient quantities of probiotics to confer benefits. However, the specific dosage may vary based on individual tolerance, study design, and the desired health outcomes, highlighting the need for personalized approaches in kefir consumption. The authors propose a dosing strategy based on body weight, suggesting a daily intake of 1.6 mL of traditionally prepared kefir per kilogram of body weight. For example, an individual weighing 90 kg would consume approximately 144 mL of kefir daily. This personalized dosing approach is designed to standardize kefir intake in clinical research, thereby facilitating a more consistent evaluation of its health-promoting effects [79].

Although kefir is generally considered safe for most individuals, research on its potential side effects or contraindications, especially in vulnerable populations like children or immunocompromised patients, is limited. One study reported a significant association between probiotic use and invasive infections with common probiotic organisms, noting differences between cases and controls [80]. Another study evaluating kefir’s safety in critically ill patients showed no kefir-related bacteremia or significant side effects, apart from diarrhea in two patients on laxatives [81]. Moslemi et al. (2024) found that daily kefir consumption significantly reduced salivary *Candida albicans* counts in chemotherapy patients without adverse effects in immunocompromised individuals [82]. Despite these findings, further studies are needed to assess kefir’s safety for vulnerable populations and identify potential contraindications. Caution should be exercised until more evidence is available.

Consumption of kefir offers oral health benefits to humans, as an effective and safe complementary anticaries strategy or periodontal treatment in the short term. Future studies like clinical trials with short- and long-term use of kefir could explain the influence of fermented beverages on dental caries and clarify which could be the most effective strains and the best way of administration. Currently, there are research groups that have provided essential information about the microorganisms and metabolites of kefir, which could be responsible for the oral health benefits, but more studies need to be performed.

Kefir is involved in the inhibition of glucosyltransferase, which encodes fructan, the binding site for *S. mutans* (A), and in the reduction in the expression levels of genes that regulate the synthesis of binding proteins (adhesin and glucan), thus preventing bacterial aggregation and biofilm formation (B and C), respectively. Factors in the development of caries such as pH control and the production of diverse metabolites are affected by kefir in the process of fermentation. (D) The figure was created with BioRender.com.

Limitations

The limitations of this study include the lack of a meta-analysis due to the heterogeneity of the samples. It is important to mention that the study’s target population was geographical areas in countries such as Iran, India, and Turkey, which belong to Asia and Europe (in the case of Turkey, both continents), respectively. Some of the reasons why these studies are only conducted in these areas may be due to cultural aspects. We mention this because the consumption of probiotic drinks is more common in ancient civilizations.

Despite the recognized potential of kefir’s probiotic properties in promoting oral health, current research reveals significant gaps that warrant further investigation. A 2023 systematic review highlighted the efficacy of probiotics in reducing oral pathogens and improving gingival and plaque indices; however, it did not specifically address kefir-based interventions, underscoring a need for targeted studies in this area [83]. Additionally, while probiotics have been suggested as beneficial for managing dental diseases, the specific strains and dosages present in kefir require detailed exploration to establish standardized therapeutic protocols. Notably, a recent clinical trial indicated that kefir consumption, as an adjunct to initial periodontal therapy, yielded improvements in periodontal clinical indices, suggesting a potential role in periodontal disease management [46].

Kefir is known for its antimicrobial and anti-inflammatory properties, but its specific role in oral health and modulating the oral microbiome requires more investigation. To address this gap, well-designed clinical trials are needed to evaluate kefir’s efficacy in oral health, especially its potential to prevent or manage dental caries and periodontal diseases. These studies could provide critical insights to inform clinical practices and public health strategies.

## 10. Conclusions

In summary, this study updates the benefits of kefir as a treatment for dental caries and periodontal disease. Kefir is a probiotic drink with minimal adverse effects and could serve as a potential prophylactic or adjuvant agent in oral health management. Future research should aim to elucidate its mechanisms of action, particularly how it modulates the oral microbiome and inflammatory pathways. Additionally, well-designed clinical trials are necessary to confirm its efficacy and explore its broader applications in dentistry.

## Figures and Tables

**Table 1:** Common oral bacteria and yeasts used for oral probiotics.

Genus	Species	References
*Lactobacillus*	*bulgaricus* *acidophillus* *casei* *paracasei* *helveticus* *lactis* *salivarius* *plantarum* *reuteri*	[30,31]
*Streptoccus*	*oralis* *uberis* *rattus* *salivarius* *thermophillus*	[30]
*Enterococcus*	*faecium* *faecalis*	[32]
*Bacillus*	*coagulans*	[30]
*Saccharomyces*	*cerevisae* *boulardii*	[33,34]

**Table 2:** Summary of Clinical Studies on Kefir.

Study Design/Country/Reference	Methodology	Dosage/Study Duration	Composition	Participants (*n*)	Relevant Results
Experimental randomized controlled study/Turkey/[43]	Participants consumed kefir. Stimulated saliva was collected to measure *S. mutans* and *Lactobacilli* counts.	100 mL and 200 mL of kefir drink per day for 3 weeks	*Lactococcus lactis* spp. *lactis*, *Lactococcus lactis* spp. *cremoris*, *Lactococcus lactis* spp. *diacetylactis*, *Leuconostoc mesenteroides* spp. *cremoris*, *Lactobacillus kefyr*, *Kluyveromyces marxianus*, *Saccharomyces unisporus*	104 volunteers (55 females, 49 males), aged 20 to 27 years	Significant reduction on counts of *S. mutans*.
Experimental, crossover randomized control trial/Iran/[16]	Group A consumed kefir. Group B used a 0.5% NaF mouth rinse, without kefir. Unstimulated saliva samples were collected before and after the intervention to measure pH levels and *S. mutans* count.	100 mL of kefir drink per day for 4 weeks	*Lactobacillus casei* subsp. *pseudo plantarum*, *Saccharomyces cerevisiae*	22 healthy volunteers (11 males and 11 females), aged 22 to 32 years	Reduction in salivary *S. mutans* being equally effective as a sodium fluoride mouth rinse. pH values show no significant difference.
Experimental, observational/India/[44]	One group consumed milk kefir, another probiotic curd, another probiotic drink, and one group was a control. Saliva samples were collected before and after both restorations and probiotic consumption.	100 mL of probiotics per day for 1 month	*Lactobacillus casei* subsp. *Pseudo plantarum*, *Saccharomyces cerevisiae*	80 healthy children, aged 8 to 12 years	Milk kefir group showed a reduction in CFUs of *S. mutans* compared to the control group in a one-month follow-up period at a weekly interval.
Experimental, randomized stratified/Turkey/[17]	One group used probiotic toothpaste; the others were the kefir group and the control group without kefir. Stimulated saliva samples were collected to measure *S. mutans* and *Lactobacillus* levels	200 mL of kefir per day for 6 weeks	*Lactococcus lactis* subsp., *Leuconostoc* sp., *Lactobacillus* sp., *S. thermophilus*, yeasts isolated from kefir grains	45 patients with orthodontic treatment aged 14 ± 2 years	Short-term consumption of kefir and use of probiotic toothpaste decreased Lactobacillus sp level compared to the control group.
Experimental, analytical cross-sectional/Iran/[45]	Participants were selected using a multistage cluster random sampling method from eight schools, combining cluster and systematic sampling. Beverage consumption frequency was recorded, and dental examinations were conducted by a WHO-trained examiner.	Unspecified dosage, 3 months of consumption	Not described	600 adolescents (300 females and 300 males) aged 12 to 15 years	A beverage frequency questionnaire was applied to the total sample where it was found that daily consumption of milk beverages with kefir decreases the DMFT index and tooth erosion.
Experimental, randomized controlled trial/Turkey/[46]	The kefir group consumed kefir in the morning without rinsing, while the control group did not consume additional supplements. Subgingival plaque samples were collected at the first and third months to measure red complex levels.	250 mL once a day during 14 days for 3 months	Not described	36 individuals in the range of 18 to 70 years	Kefir improved clinical and microbiological outcomes in periodontitis patients, similar to other probiotics. No significant differences were found in baseline (T0) clinical indexes.
